# Opportunistic osteoporosis screening on FDG PET/CT scans in breast carcinoma: a comparison with DXA

**DOI:** 10.22038/aojnmb.2025.86503.1619

**Published:** 2026

**Authors:** Nitin Gupta, Manpreet Kaur

**Affiliations:** 1 Department of Nuclear Medicine, Dr Rajendra Prasad Government Medical College and Hospsital , Kangra Himachal Pradesh India; 2 Department Nuclear Medicine, Post Graduate Instititute of Medical Education and Research Chandigarh India

**Keywords:** Breast carcinoma, FDG PET/CT, Hounsfield units, DXA scan, Osteoporosis, Fracture risk assessment A B S T R A C T

## Abstract

**Obgective(s):**

Reduced bone mineral density is often observed in breast cancer patients. Routine PET/CT scans can be used for detection of low bone mineral density. To evaluate prevalence of osteopenia, osteoporosis and fracture risk in pre and post-therapy breast carcinoma patients undergoing ^18^F-FDG PET/CT scans.

**Methods:**

In this retrospective study L1-L4 vertebral and femoral neck CT mean Hounsfield unit attenuation and their corresponding SUV_max_ values from initial staging and end of treatment FDG PET/CT scans performed in breast carcinoma patients were compared. Post chemo ± hormonal therapy FDG PET/CT HU values were also compared to DXA scan T- scores.

**Results:**

Significant increase in prevalence of post chemo ± hormonal therapy osteopenia, osteoporosis and fractures (62%, 18% and 16% vs baseline of 35%, 4% and 9% respectively). CECT mean attenuation values of ≤174.6 HU and ≤117.2 HU for detection of osteopenia and osteoporosis with sensitivity of 100% and specificity of 94.2 % for L1-L4 vertebrae, and ≤176.8 HU and ≤117.8 HU for osteopenia and osteoporosis with sensitivity of 100% and specificity 96.4% at femoral necks respectively were suggested. Furthermore, mean attenuation values of ≤125.9 HU with sensitivity and specificity of ~100% and 79% and ≤124.8 HU with sensitivity of 100% and specificity ~79.8% were suggested for increased L1-L4 vertebral and femoral neck fractures prevalence/ risk respectively. An associated post chemo ± hormonal therapy decline in vertebral and femoral neck mean SUV_max_ values in range of 14% was also observed.

**Conclusions:**

Baseline and post chemo ± hormonal therapy follow up FDG PET/CT scans allow opportunistic evaluation and can identify a significant number of patients with osteopenia, osteoporosis and patients at increased fracture risk with a high sensitivity and good specificity. They have potential to reduce need for DXA referrals, and also enable early initiation of prophylasix and therapy.

## Introduction

 Osteoporosis is characterized by reduced bone mineral density and microarchitectural deterioration of bone tissue, leading to increased susceptibility to fractures (1). Osteopenia refers to bone density that is not normal but also not as low as osteoporosis World Health Organization defines osteopenia by bone densitometry as T score of -1 to -2.5 (2). Varying degrees of treatment related bone loss has been reported in postmenopausal breast cancer survivors (3-5). Cody et al (6) suggested that younger breast cancer survivors are also at higher risk of osteoporosis compared to cancer free patients. 

 Currently DXA scan (dual energy X-ray absorptiometry) is considered as gold standard method for assessment of BMD (3). However, several studies have found that DXA scan may yield a false negative result due to misinterpretation of BMD of lumbar spine because of various confounding factors (7). 

 Degenerative osteophytes, endplate sclerosis, facet arthropathy, aortic/iliac calcifications, vertebral compression deformities, and focal sclerotic lesions can artifactually elevate a real BMD and yield falsely high Tscores, masking osteopenia/osteoporosis. DXA also cannot separate trabecular from cortical bone and is sensitive to positioning and body habitus, which are frequent issues in patients receiving systemic therapy. As a result, a substantial proportion of fragility fractures occur in individuals categorized as osteopenic rather than frankly osteoporotic on DXA, underscoring the need for complementary risk stratification beyond DXA alone (8-10).

 Some studies in literature have demonstrated that vertebral and femoral neck attenuation in Hounsfield units (HU) from abdominal-pelvic CT scans performed for other indications can be used for opportunistic osteoporosis screening (11-13). Furthermore, CT derived HU attenuation values could be used to predict future osteoporotic fracture risk (14, 15). Another advantage of using CT based HU attenuation is that they do not vary based on sex, ethnicity, age, height, or weight (16).

^18^F-FDG PET/CT is widely used for baseline and follow-up imaging in breast carcinoma patients. Few studies have reported assessment of BMD from ^18^F-FDG PET/CT scans in lymphoma patients (17, 18)_. _Therefore, we undertook this study to assess utility of ^18^F-FDG PET/CT for opportunistic screening of osteopenia/osteoporosis and increased fracture risk assessment in breast carcinoma patients.

## Methods

 This retrospective study included analysis of clinical data of patients with biopsy confirmed breast carcinoma and their initial staging ^18^F-FDG PET/CT scans (PET/CT#1) and subsequent response assessment/follow up FDG PET/CT scans (PET/CT#2) Mean interval between PET/CT#1 and PET/CT #2 was 7 months. Vertebral HU (VHU) and femoral neck HU (FNHU) values in PET/CT #2 were also correlated with respective post chemo ± hormonal therapy DXA scan T- scores. Mean interval between PET/CT #2 and DXA scans was 5 weeks.

 Patients with vertebral metastasis and/or bone marrow involvement, known history of severe compression fracture or history of compression fracture surgery, hematological disease, known metabolic bone disease, or chronic kidney disease were excluded from the study.

 Patients were classified into two categories; A) Younger women (age range between 25-46 years) and (B) post-menopausal women: age ≥47 years.

^18^ ***F-FDG PET/CT Acquisition***

 All PET/CT scans had been acquired on a Discovery STE 64 slice PET/CT scanner (General Electric Company, Milwaukee, WI, USA) according to institutional protocol. Images were reconstructed using 1.5 mm thickness using bone kernel and 512×512 image matrix. 

 Intravenous contrast increases trabecular attenuation at the lumbar spine (typically in the range of ~10-25 HU above non contrast values, depending on phase and patient factors) and has a smaller relative effect at the proximal femur (often only a few percent). To minimize technical variability we (i) excluded scans obtained at tube voltages other than 120 kVp, because tube voltage has been shown to significantly affect vertebral attenuation (19), (ii) analyzed vertebral and femoral neck regions separately, and (iii) interpreted our HU thresholds explicitly in the context of contrast enhancement. Consequently, the ROC derived cut points for osteopenia/osteoporosis and fracture risk in this study are expectedly higher than published non contrast thresholds. 

***Vertebral, femoral neck CT attenuations, SUV***_max_ ***measurements, criterion and interpretation***

 Retrospective manual measurements of mean CT attenuation of trabecular bone in L1-L4 lumbar vertebrae (mean VHU) and bilateral femoral neck in Hounsfield units (FNHU) were performed on a standard picture archiving and communication system workstation. 

***ROI/VOI placement and measurement protocol***

 On the CT component, a reader with 10 years PET/CT experience placed elliptical or circular ROIs entirely within trabecular bone, avoiding cortical shell, endplates, Schmorl nodes, hemangiomas, hardware, and focal sclerosis. 

 For L1-L4, a target ROI area of 150-250 mm² was used, centered in the vertebral body on three contiguous axial slices per vertebra; the three values were averaged per level, and levels were then averaged to yield mean VHU. Any vertebra with compression/collapse, extensive degenerative change precluding a clean trabecular sample, or focal lesion was excluded a priori from the L1-L4 mean; the mean was computed from the remaining levels (minimum two levels required). For the femoral neck, bilateral elliptical ROIs of 80-120 mm² were placed at the mid-neck, avoiding subcortical bone and osteophytes; left/right values were averaged for mean FNHU. CT window/level was standardized (e.g., W: 2000, L: 300) and ROIs were copied between time points when anatomy

permitted to reduce placement variability.

 On the PET component, a spherical VOI (diameter 10-15 mm) was centered on the corresponding trabecular regions at each vertebral level and mid femoral neck bilaterally to obtain SUV_max_. VOIs were carefully positioned to avoid adjacent metabolically active structures (e.g., cortical bone, endplates, vessels). For each patient and time point, vertebral VOI SUV_max_ values were averaged (L1-L4) to yield mean vertebral SUV_max_, and femoral VOI SUV_max_ values were averaged bilaterally for mean femoral neck SUV_max_. All measurements were performed twice in a subset of 20 randomly selected cases to confirm reproducibility.

***Quality control and discrepant regions***

 If large discordance existed between vertebral and femoral measurements (e.g., lumbar osteopenia with normal femoral neck), both values were retained and reported, as such patterns occur biologically and may have management implications. 

**Figure 1:**
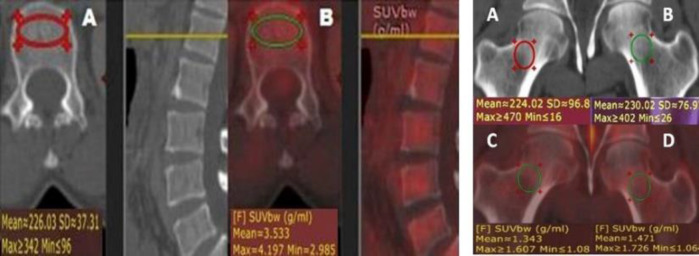
Examples of methods of calculation of vertebral and femoral neck CT HU attenuation and SUV_max_ measurements

 Mean L1-L4 attenuation (mean VHU), mean femoral neck attenuation (mean FNHU), and corresponding mean SUV_max_ values were measured as shown (Figure 1)**. **

 For FDG PET/CT based patient classification into normal, into osteoprotic and osteopenic categories, predetermined CECT (contrast enhanced computed tomography) mean vertebral attenuation values of below 118 HU, and between 118 and 178 HU defined respectively based on data from some of the previous studies (13, 20-22). Furthermore, mean VHU less than 90 HU was used to determine risk for osteoporotic vertebral fracture and decreased fracture free survival (14, 15). 

 FNHU of 70±30 HU for osteoporosis, 110±36 HU for osteopenia and 158±43 HU for normal BMD were used based on data from a previous study (20). PET/CT#2 mean VHU and FNHU values were compared to DXA L1-L4 reference T-scores and mean femoral neck T-scores respectively.

***DXA scans***

 DXA scans were performed on GE Lunar iDXA scanner (GE Healthcare, Waukesha, WI). DXA L1-L4 reference and mean bilateral femoral neck T-scores were recorded. Subjects were categorized as having osteopenia (DXA T-score between -1.0 and -2.5 SD), osteoporosis (DXA T-score ≤ -2.5 to -3.0 SD) and severe osteoporosis (DXA T-score < -3.0 SD). 

***Statistical Analysis***

 All statistical analysis was carried out using MedCalc (version 23.0.9, MedCalc Software Ltd, Ostend, Belgium) and Prism GraphPad (version 10.4.0, GraphPad Software LLC.) statistical softwares. Patient demographic and clinical characteristics were described using mean, standard deviation (SD), and range, for continuous variables and as frequency and percentage for categorical variables. Normalcy of data were checked using Shapiro-Wilk and Lilliefor’s tests. None normally distributed data was normalized using Box and Cox log transformation tool.

 Preliminary distribution checks (Shapiro-Wilk and Lilliefors) indicated skewness and variance non uniformity for HU and DXA variables, particularly in post therapy data. To better satisfy assumptions for linear correlation and agreement analyses (Pearson correlation, Passing-Bablok regression, and Bland-Altman limits of agreement), we applied a Box-Cox family transformation to HU and DXA measurements. The transformation parameter λ was estimated by maximum likelihood on the training dataset; values near λ=0 implied a log transform. Box-Cox preserves rank order while stabilizing variance and improving linearity, there by yielding more reliable confidence intervals for bias and proportional differences. For robustness, key comparisons were also evaluated with non-parametric methods (Wilcoxon tests; Spearman correlation), which led to the same substantive conclusions.

 Mean VHU, FNHU and SUV_max_ values from pre and post therapy scans respectively were compared using Wilcoxon test for paired samples. A mean HU attenuation decline of >15% was considered significant. Correlations of mean VHU values and FNHU with mean SUV_max_ values respectively were also analyzed using Pearson’s correlation coefficient. P value 

< 0.05 was considered as as significant. Bland-Altman analysis plots and Passing and Bablok regression analysis were used for comparison and analysis of transformed mean VHU, mean FNHU and DXA T-scores. Spearman’s rank correlation coefficients were also calculated. Grubbs's double sided test was used to detect any outliers in data sets.

 ROC curve analysis with Youden J index were also carried out to find mean VHU and FNHU for 

detection of osteopenia, osteoporosis. ROC analysis was also performed to identify threshold for increase fracture risk frequency/tendency. Mean VHU and FNHU related relative risk and Odds’s ratio for vertebral and femoral neck fractures were determined. In addition sensitivity, specificity, positive predictive value and negative predictive values of mean VHU and FNHU values were also determined for increased fracture risk.

## Results

 This retrospective study included 114 women with newly diagnosed breast cancer selected as per aforementioned criterion and treated with standard first-line chemotherapy and hormonal regimens. Mean patient age was 39 years (age range: 27- 85 years).

**Figure 2:**
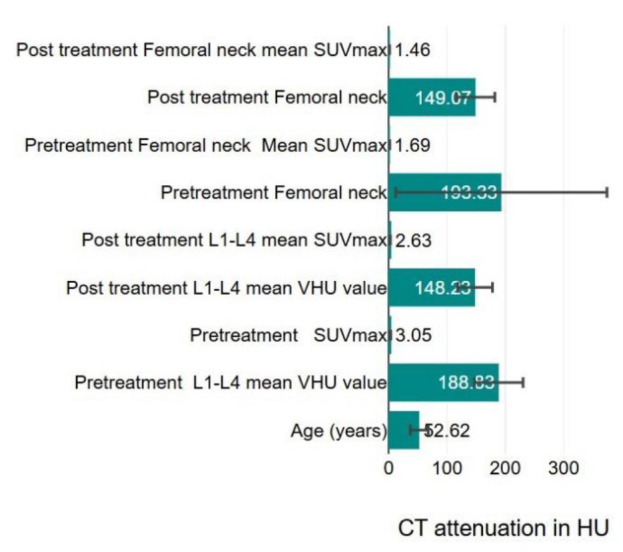
Bar Chart depicting mean patient age, pre and post treatment L1-L4 and femoral CECT mean attenuation values in HU and corresponding SUV_max_ values with black lines showing standard deviations

 PET/CT#1 and PET/CT#2 scan derived mean VHU, FNHU and corresponding SUV_max_ values are depicted in Figure 2. Further, age decade group wise patient data is depicted in Tables 1 and 2. 

**Table 1:** Age group wise representation of pre and post chemo ± hormonal therapy mean L1-L4 VHU values and corresponding mean SUV_max_ values

Age group (years)	PET/CT#1 mean VHU		PET/CT#2 mean VHU		PET/CT#1 mean SUV_max_		PET/CT#2 mean SUV_max_	
	Mean	Range	Mean	Range	Mean	Range	Mean	Range
**81-90 (n=3)**	103.1	(88-113.4) HU	107	(87.2-121.6) HU	1.63	(1.42-1.79)	1.50	(1.3-1.6)
**71-80 (n=15)**	144.4	(124-182) HU	114.7	((92.4-133.2) HU	2.42	(1.89-3.3)	2.07	(1.78-2.78)
**61-70 ( n=16)**	160.7	(60.1-206) HU	128.9	(106.7-161.4) HU	2.72	(2.38-3.54)	2.38	(2.1- 3.1)
**46-60 (n=44)**	86.5	(48-225) HU	144.9	(94.3-160.4) HU	2.96	(1.98-4.73)	2.60	(2.15-3.95)
**35-45 (n=23)**	15.3	(147.6-245) HU	166	(108.9-190.7) HU	3.33	(2.86-4.49)	2.89	(2.11-3.37)
**24-34 (n=19)**	237.2	(154.2-267.4) HU	185.5	(103-216.4) HU	3.92	(3.1-5.13)	3.33	(2.17-4.38)

**Table 2:** Age group wise representation of pre and post chemo ± hormonal therapy mean FNHU values and corresponding mean SUV_max_ values

Age group (years)	Pre treatment FNHU		Post treatment FNHU		Pre treatment SUV_max_		Post treatment SUV_max_	
	Mean	Pt. Range	Mean	Range	Mean	Range	Mean	Range
**81-90 (n=3)**	83.2	(74.8-91.4)HU	79.9	(54.8- 108.6) HU	1.33	(1.23-1.46)HU	1.0	(0.92-1.15)
**71-80 (n=15)**	134.7	(102.4-188.2) HU	109.2	(71.2-170.4) HU	1.6	(0.86-1.96)HU	1.32	(0.72-1.78)
**61-70 (n=16)**	151.5	(76.4-190.4) HU	128	(97.6-164.6) HU	1.55	(0.82 -2.2)HU	1.41	(0.92-1.96)
**60-46 (n=44)**	179.3	(56.8-225.3) HU	154.8	(84.6-198.6) HU	1.65	(0.94-2.47)HU	1.44	(0.76-1.94)
**35-45 (n=23)**	198.4	(138.8-235) HU	162.7	(107-190.7) HU	1.78	(1.16-2.5)HU	1.56	(1.1-2.1)
**25-34 (n=19)**	219.1	(141- 252.7) HU	185.5	(114.2-212) HU	1.88	(1.34-2.82)HU	1.6	(1.1-2.4)

On pre chemo ± hormonal therapy (PET/CT#1) scans, mean VHU <118 HU was observed in 4 (~3%) patients whereas mean VHU between118-178 was seen in 35 (~31%) patients compared to post treatment (PET/ CT#2) scan mean VHU < 118 HU in 18 (~16%) patients and between 118-178 HU in 75(~65%) patients.

**Figure 3:**
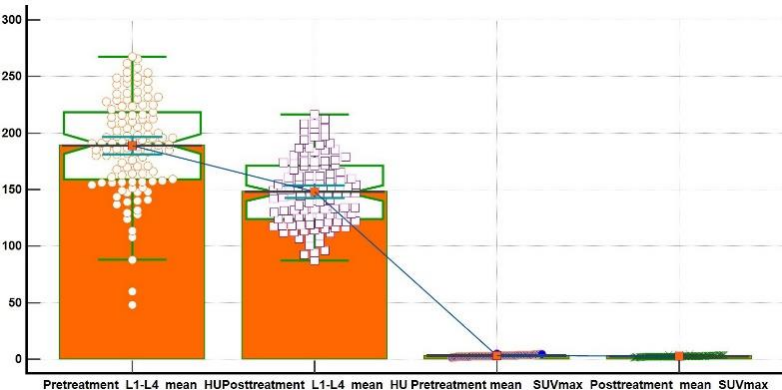
Wilcoxon test for paired samples depicting distribution and differences of pre and post chemo ± hormonal therapy L1-L4 VHU and SUV_max_ values

**Figure 4:**
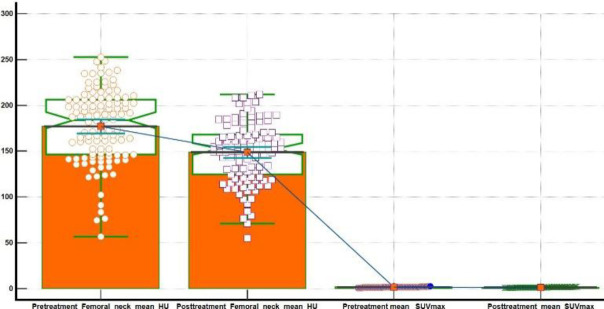
Wilcoxon test for paired samples depicting distribution and differences of pre and post chemo ± hormonal therapy FNHU and SUV_max_ values. Number of Positive differences was 3 while of that negative differences was 111 with test statistic Z = - 8.61 and two tailed probability value, p<0.0001

 Wilcoxon test for paired samples was used to assess statistical differences between PET/ CT#1 and PET/CT#2 mean VHU and FNHU values and their corresponding SUV_max_ values respectively (Figures 3, 4). Hodges-Lehmann median difference of - 42.8 HU (95% C.I. -45 to -40.7 HU) was observed between pre and post chemo ± hormonal mean VHU values, with test Z statistic value of -8.8 at two tailed probability P value < 0.0001. For FNHU, a Hodges-Lehmann median difference of - 28.8 HU (95% C.I. - 31 to - 25 HU) with a test Z statistic value of -8.5 at two tailed P value=0.0001 was observed. An average percent decline ~21.5% (range from 8.2% to 30%) was observed in between pre and post chemo ± hormonal mean VHU and FNHU values and a percent decline of ~14% in mean SUV_max_ values was seen.

**Figure 5:**
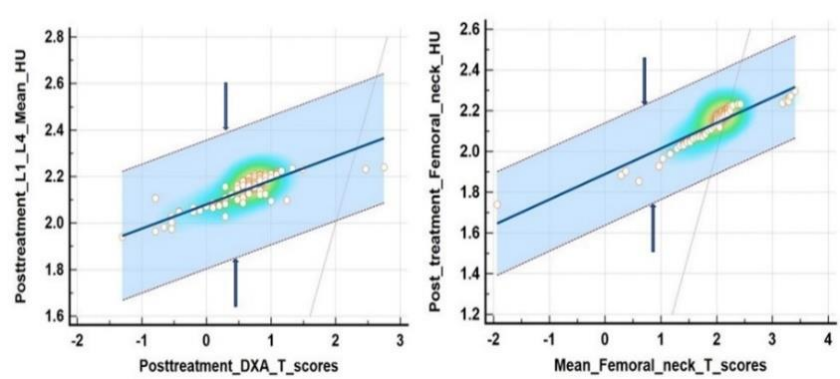
Scatter plots along with heap map of post chemo ± hormonal therapy Box Cox log transformed mean VHU and mean FNHU values vs respective DXA reference T- scores respectively. Central black line represents line of equality whereas two lined marked with blue arrows on it’s either side represent 95% confidence limits

 Scatter plots along with heap map of post chemo ± hormonal therapy Box Cox log transformed CT mean VHU and mean FNHU values vs respective DXA reference T- scores respectively are depicted in Figure 5.

**Figure 6:**
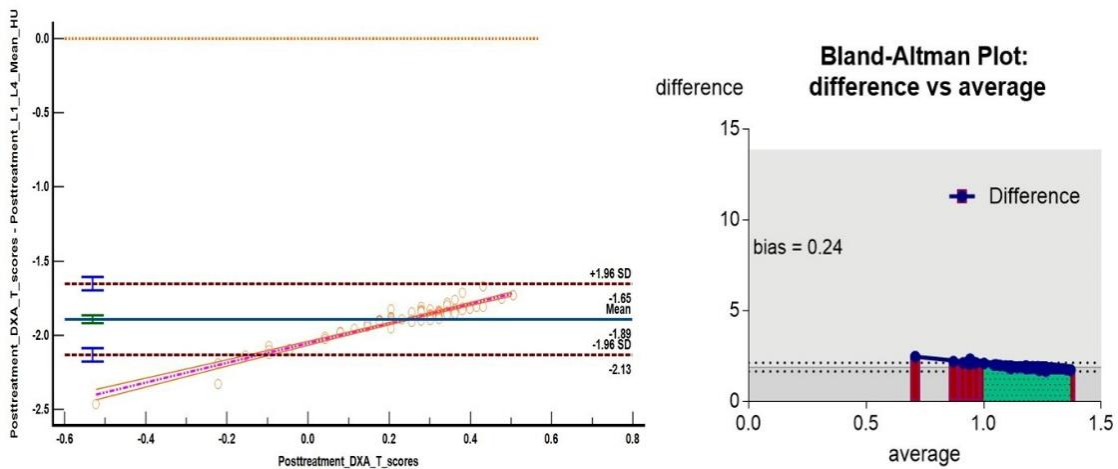
Bland - Altman plot between Box Cox log transformed post chemo ± hormonal therapy mean VHU values and mean DXA T-scores. Brick-red dotted lines show 95% confidence limits of agreement

**Figure 7:**
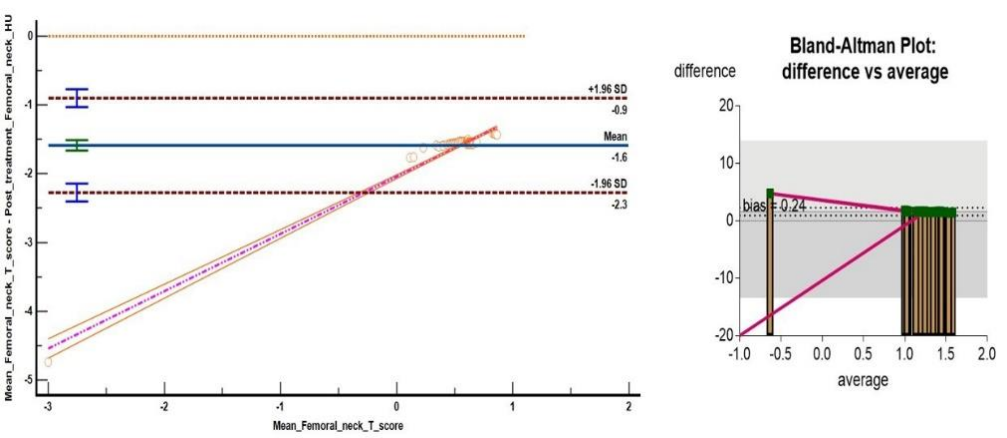
Bland - Altman plot analysis between Box Cox log transformed post chemo ± hormonal therapy femoral neck mean DXA T-scores and mean FNHU values. Brick-red dotted lines show 95% confidence limits of agreement

**Figure 8:**
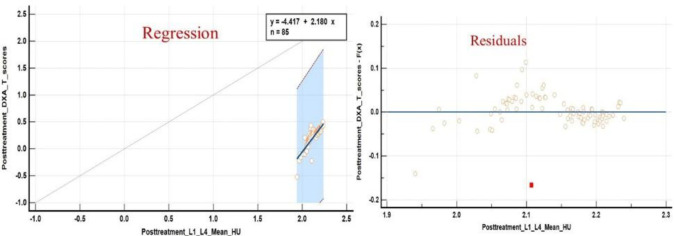
Passing and Bablok regression analysis between Box Cox log transformed L1-L4 mean HU and DXA T- scores. Analysis shows a systemic difference of -1.84 (95% C.I. -1.6 to -2.1) and proportional difference of 1.1 (95% C.I. 1.02 to 1.25) indicating minor differences between measurements obtained from two techniques

**Figure 9:**
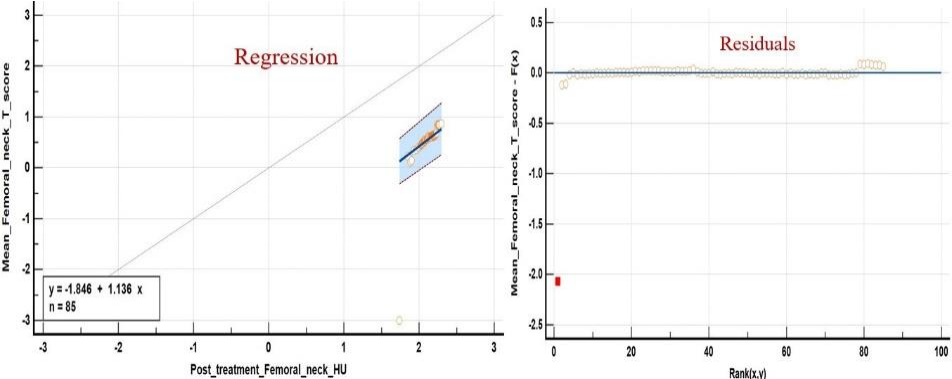
Passing and Bablok regression analysis between Box Cox log transformed FNHU and DXA T- scores. Analysis shows a systemic difference of -1.84 (95% C.I. -1.6 to -2.1) and proportional difference of 1.1 (95% C.I. 1.02 to 1.25) indicating minor differences between measurements obtained from two techniques

We found a significant correlation between PET/CT#2 mean VHU, mean FNHU and respective DXA mean T- scores after Box-Cox log transformation using Bland Altman plots (Figures 6, 7) and Passing and Bablok regression analysis (Figures 8, 9). L1-L4 mean T- scores ranged from -3.8 to -1.0 corresponding to mean L1-L4 VHU of 87.2 and 174.2 HU respectively (median T- score of -2.0 corresponding to VHU of 143.2 HU). 

 Spearman’s rank correlation coefficient was 0.703 (95% C.I.; 0.632 to 0.774) with p value <0.0001. No significant deviation from linearity was observed (p=0.10). For femoral neck, mean T- scores ranged from -5.9 to 1.4 SD corresponding to mean FNHU of 54.8 and 198 HU respectively (median T-score of -1.6 SD corresponding to FNHU of 156 HU); Spearman’s rank correlation coefficient between two modalities was observed as 0.814 (95% C.I. 0.726 to 0.890) with p value <0.0001. 

 Results of ROC analysis suggested balanced thresholds of L1-L4 mean CT attenuation value ≤ 174.6 HU (95% C.I. of ≤172.4 to ≤174.6 HU) and ≤ 117.2 HU ( 95% C.I. of ≤ 116 to ≤ 117.2 HU) for detection of osteopenia and osteo-porosis respectively with a sensitivity of 100% and specificity ~ 94.2% , with AUC 0.905 at P value <0.001, while an optimum criteria ≤116 HU ( 95% C.I. of ≤112 to ≤117.6 HU) for detection of osteoporosis with sensitivity of 92.8% and specificity ~100%, whereas a femoral neck mean attenuation ≤176.8 HU (95% C.I. of ≤174.2 to ≤176.8 HU) for detection of osteopenia and ≤117.8 HU ( 95% C.I. of ≤116.6 to ≤117.8 HU) for detection of osteoporosis with sensitivity of 100% and specificity ~ 98.4%; and Youden J index of 0.984 at P value <0.001 on contract enhanced PET/CT scans.

**Figure 10:**
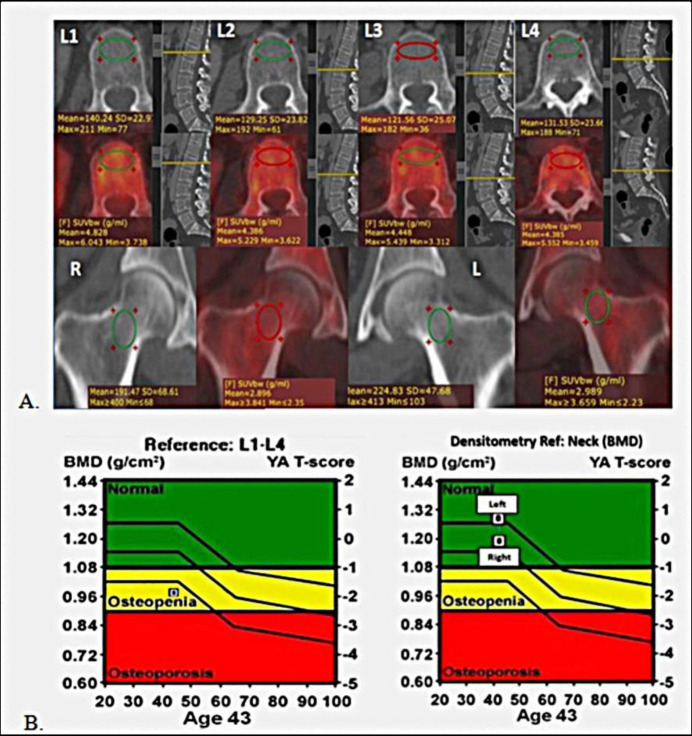
A 43 year old breast carcinoma patient, follow up PET/CT was done 7 months after Chemo ± hormonal therapy. Axial CECT (top row) shows significantly reduced L1-L4 HU (Mean CT attenuation ~130.6HU),whereas FNHU values were within normal range (mean FNHU ~208.1HU). Respective mean DXA T-scores were -1.9 and -0.2 for L1-L4 and femoral necks. Pre chemo ± hormonal therapy DXA was not done. This case also demonstrates that there can discrepancy between vertebral and femoral neck BMD and hence both should be screened on routine PET/CT scans in breast carcinoma patients

 Some of the patients showed significant discrepancy between mean attenuation values of lumbar vertebrae and femoral necks with lumbar vertebrae being osteopenic while femora having normal attenuation (example Figure 10) or vice versa. 

**Figure 11:**
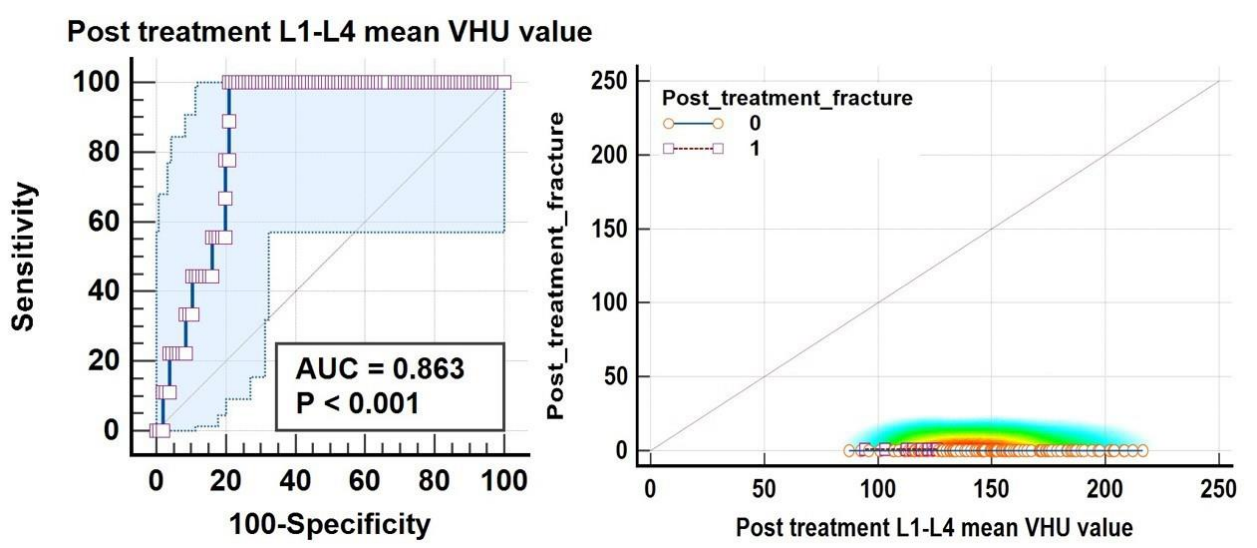
ROC curve analysis along with concordance correlation coefficient scatter charts for increased fracture frequency and fracture risk at L1-L4 vertebrae suggested a balanced threshold value of ≤125.9 HU whereas an optimal criteria of VHU ≤87 HU

**Figure 12:**
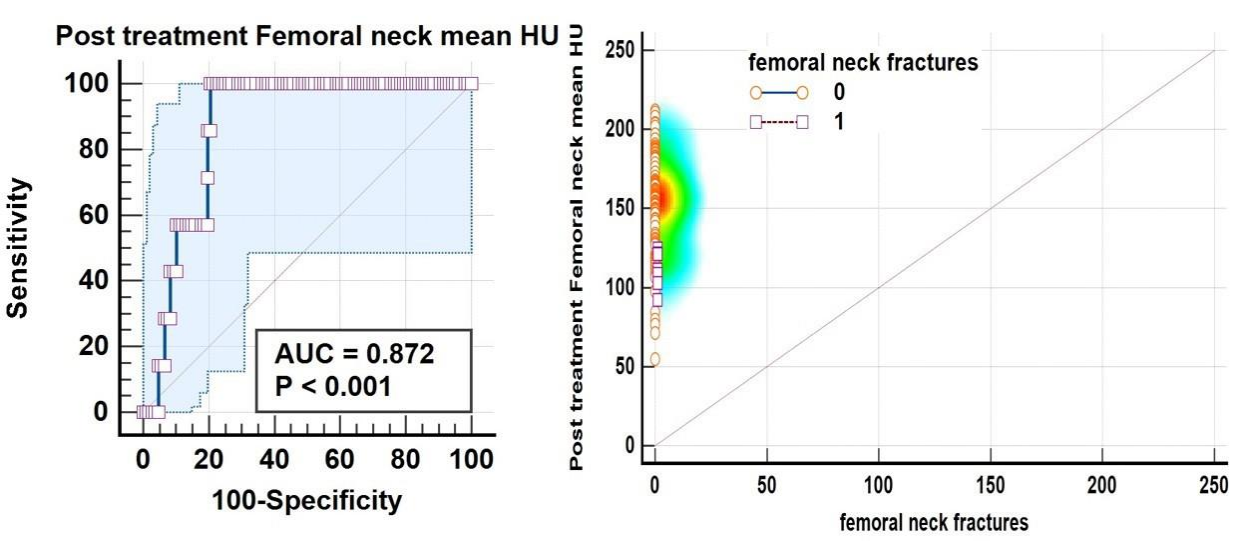
ROC curve analysis revealed increased fracture frequency/ risk at mean FNHU attenuation ≤124.8 HU with a sensitivity of 100% and specificity ~79.8% and a threshold ≤54.8 HU with 100% specificity

ROC analysis also suggested a balanced threshold value of ≤125.9 HU (95% C.I. of ≤123.8 to ≤125.9 HU) with Yuoden J index of 0.790, sensitivity and specificity ~100% and 79% with standard error of 0.035 at P value <0.0001; while at optimal criteria of VHU ≤87 HU (95% C.I. ≤87.2≤94.3 HU) 100 % specificity was observed for vertebral fractures (Figure 11) whereas a mean attenuation ≤124.8 HU (95% C.I: ≤121.6 ≤124.8 HU) with sensitivity of 100% and specificity~79.8% with Youden index J ~0.794 and AUC of 0.872; standard error of 0.037 at P value <0.0001 for increased femoral neck fracture prevalence/ risk (Figure 12).

**Figure 13:**
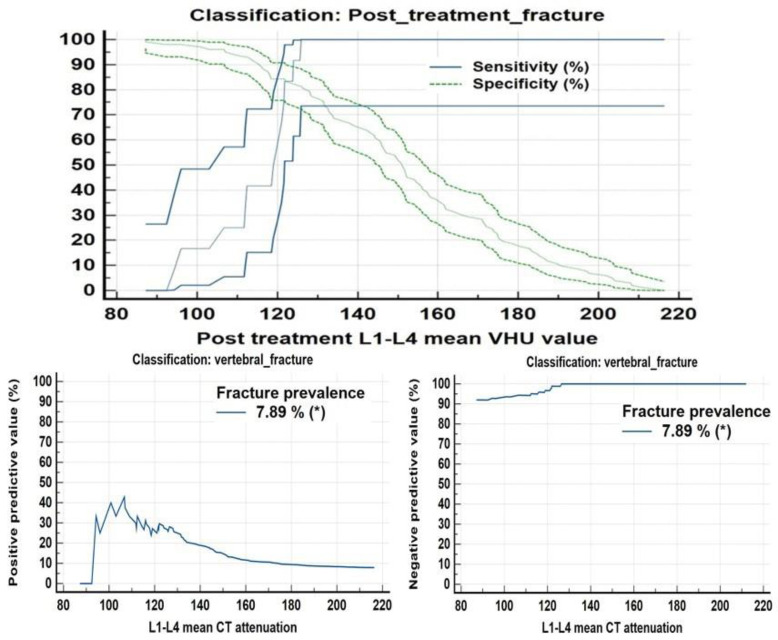
Criterion Plots showing at mean CECT VHU ~125.9, sensitivity specificity, PPV and NPV for vertebral fractures were 100%, 79%, 38.2% and 100% whereas at mean VHU ~97HU, values were 27.2%, 99.2%, 79.9% and 92.1% respectively. At a mean VHU≤ 87 HU specificity was ~100%

**Figure 14:**
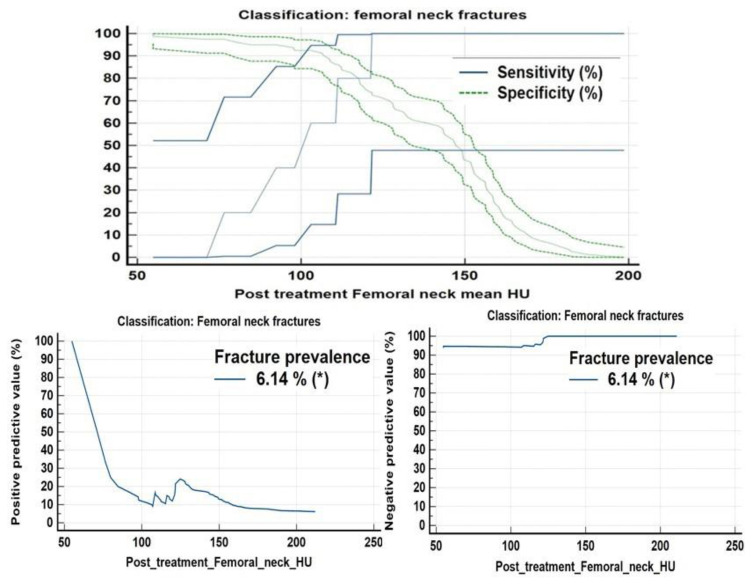
Criterion plots for mean FNHU for predicting/indentifying increased fracture frequency/ risk. At mean FNHU attenuation ~124.8 HU, sensitivity of 100%, specificity~ 79.8%, PPV, and NPV of 31% and 100% respectively, while at mean FNHU ~54.8 HU, sensitivity of ~ 11.1%, and Specificity ~100% was observed

Figures 13, 14 present fracture prevalence, sensitivity, specificity, positive and negative predictive values of mean attenuation values for increased fracture risk at L1-L4 vertebrae and femoral necks respectively. A comparison of post chemo ± hormonal vertebral and femoral neck CT mean attenuation values and DXA T- scores is shown in Tables 3 and 4.

**Table 3:** Comparison of post chemo ± hormonal therapy mean VHU, SUV_max _values and DXA T-scores between younger and post-menopausal breast carcinoma patients

**Group category**	**Age range**	**No. of Patients**	**Post- chemo ± hormonal therapy mean L1-L4 VHU**		**Post chemo ± hormonal therapy DXA Reference mean L1-L4 T - Scores**	
Young women	25-46 years	N= 44	Osteopenia	Osteoporosis	Osteopenia	Osteoporosis
			N=21	N=3	N=17	N=2
			**Mean** **VHU / SUV**_max_	**Mean ** **VHU / SUV**_max_	**Osteopenic ** **T- score range**	**Osteoporotic ** **T- score range**
			159.8/3.0	108.7/2.18	-1.0 to -2.2	-2.6 to -3.1
Post-menopausal	47-85 years	N= 70	N=55	N=15	Osteopenic T- score Range	Osteoporotic T- score range
			140.4/2.47	106.6/2.0	-1.2 to -2.4	-2.6 to -3.8

**Table 4:** Comparison of post chemo ± hormonal therapy mean FNHU, SUV_max_ values and DXA T-scores between younger and post-menopausal breast carcinoma patients

**Group category**	**Age range**	**No. of Patients**	**Post chemo ± hormonal therapy FNHU**		**Post chemo ± hormonal therapy DXA Femoral neck mean T - Scores**	
Young women	25-46 years	N=44	Osteopenia	Osteoporosis	Osteopenia	Osteoporosis
			N=22	N=2	N=17	N=2
			**FNHU/ SUV**_max_	**FNHU / SUV**_max_	**T- score Range**	**T-score range**
			170.4/1.58	110.6/2.1	-1.1 to -2.4	-2.6 to -2.8
Post-menopausal	47-85 years	N=70	N=46	N=19	N=39	N=17
			145.8/1.46	99.2/1.9	-1.2 to -2.3	-2.7 to -5.6

 On PET/CT #1 scans, fractures were detected in 10 (~9%) patients, including vertebral compression fractures in five patients, femoral neck fractures in three patient and rib fracture in two patients, whereas on PET/CT #2 scans, fractures were detected in 19 (~16%) patients including nine vertebral compression fractures, seven patients with femoral neck fractures, rib fractures in two, and pubic ramus fracture in one patient. Paired T test showed statistically significant difference for fracture prevalence between PET/CT #1 and PET/CT #2 scans with two tailed P value of 0.01. Relative risk and Odd’s ratio for post chemo ± hormonal therapy fractures were 1.8 (95% C.I. 1.68-1.98); 1.87 (95% C.I. 1.7-2.2) and 2.3 (95% C.I. 2.06 to 2.6) and 2.42 (95% C.I. 2.12 to 2.75) for vertebral and femoral neck fractures respectively with P values <0.001. 

## Discussion

 In our study, 35(~31%) and 5(~4%) women were found to have osteopenia and osteoporosis respectively at baseline/staging PET/CT. In a retrospective analysis by Kay Fu et al (18), authors demonstrated osteoporosis and osteopenia in 16% and 46% of patients≥ 50 years old respectively among 100 patients on staging ^18^F-FDG PET/CT scans. Some studies (21-23) have reported an overall prevalence of osteopenia and osteoporosis in Indian women at ~18.3% and ~49.9% respectively. Because all scans in our study included portal venous phase CECT at 120 kVp, the observed HU thresholds for osteopenia/osteoporosis and fracture risk represent contrast adjusted values and should not be directly equated to non-contrast benchmarks commonly cited from abdominopelvic CT cohorts.

 There was median 42.8 HU decline (an average percent decline ~21.5%) and 28.8 HU decline (an average percent decline ~23%) between pre and post chemo ± hormonal mean VHU and FNHU values respectively in our study. At baseline (PET/CT#), 35(~31%) and 5(~4%) women were found to have osteopenia and osteoporosis respectively compared to 71(~62%) and 21 (18%) patients with osteopenia and osteoporosis respectively on post chemo ± hormonal (PET/CT #2) scans. In comparison, Ofshenko et al (24) documented a decline of 16.4±11.2% from baseline in L1-L4 mean VHU at end of treatment scan. At baseline, thirty eight patients (32%) had osteopenia and three (~2%) patient had osteoporosis whereas post therapy FDG/PET scans revealed osteopenia in sixty one (~52%) of patients and osteoporosis in nine (~7%) of patients.

 In another study, Cohen et al (17) found a mean 14.2% decline in VHU at end of chemotherapy PET/CT #2 scans compared to baseline PET/CT #1 in lymphoma patients. Twelve patients (~15%) had abnormal BMD on PET/CT #2, compared to nine patients (~11%) on PET/CT #1. They concluded that available PET/CT scans may obviate the need for further DXA scans and have potential to detect patients at high risk for developing osteoporosis. 

 Most common causes of bone loss in women are aging and menopause. Aging is associated with greater bone resorption and less bone formation, whereas menopause induces accelerated bone loss due to lowering levels of endogenous estrogen (25). In a review of studies, average age at menopause for India was found to be 46.6 years (95% CI: 44.83 to 48.44) (26). Therefore we used age limit of 47 years to define post-menopausal group among our study sample.

 Chemotherapy may cause bone loss due to treatment induced premature menopause and may also have direct toxic effects on osteoblasts (27, 28). Breast cancer patients treated with aromatase inhibitors, chemotherapy regimens plus aromatase inhibitors or selective estrogen receptor modulators such as Tamoxifen have been reported to have a greater than two to three times increased risk of osteopenia and osteoporosis as compared to cancer free women (6). Some studies have suggested that tamoxifen may cause bone loss among premenopausal women by inducing premature menopause (29, 30). Further, glucocorticoids administered prior to and following chemotherapy can lead to osteopenia and even osteoporosis (31-33). In line with these aforementioned studies, present study also demonstrated a significant decline in BMD measured indirectly via mean VHU and FNHU values in breast cancer patients treated with chemo ± hormonal therapy. 

 Currently, DXA is considered as gold standard method for BMD assessment (6). However, several studies have demonstrated that degenerative changes, compression fractures, sclerotic lesions, vascular calcifications and increased body fat may result in spuriously high measurement of BMD (11, 12). In addition, DXA suffers from inability to discriminate between cortical and trabecular bone mass (11). A study by Krueger et al (7) reported technical errors in 90% of patients while interpretation errors were present in 80% of patients; of which 42% were major. Further, DXA T-scores alone indicating osteopenia or osteoporosis, do not necessarily mean that a fracture will ensue. 

 Majority of patients who suffer fracture have osteopenia rather than osteoporosis as defined by DXA T-scores (10) Relying on T- scores from DXA alone can be problematic. This is because there are several non-skeletal factors that impact fracture risk. Therefore a clinical assessment tool FRAX® calculator, is used to integrate other partly BMD independent risk factors with BMD on DXA (34).

 Some studies in literature have documented that CT VHU values during abdomen or thorax CT scans can be used for opportunistic osteoporosis screening (11-13). Studies in literature have reported NCCT VHU values below 100 HU as indicative of osteoporosis, between 100 and 160 HU indicative of osteopenia, and values above 160 HU as normal bone mineral density. Further NCCT VHU less than 90 HU has been documented as a risk for osteoporotic vertebral fracture and significantly associated with decreased fracture free survival (11-13). 

 For FNHU values, Christensen et al (20) reported mean comprehensive NCCT HU for osteoporosis as 70±30 HU, 110±36 HU for osteopenia and 158±43 HU for normal BMD (p<0.001), while in study by Yazar Isimleri (35), DXA classified average FNHU of 178 HU as normal, ~117 HU as osteopenia and average NCCT attenuation <60 HU as osteoporosis with P value <0.01. 

 However, some studies have documented a median difference of 16 HU (12.5-23.5) in vertebral attenuation between unenhanced phase and portal phase after administering contrast agent (36, 37). Therefore, if attenuation measurements are not adjusted for effect of contrast enhancement, contrast enhanced CT can result in under diagnosis of osteoporosis in 7-25% of patients. In contrast, post contrast bone attenuation values increase only approximately 2% in proximal femur (38). 

 There are a few studies in literature which have reported assessment of BMD from routine oncologic FDG PET/CT scans (17, 18). However, these studies were based on FDG PET/CT scans without use of intravenous contrast. 

 In contrast, since all patients in our study had undergone contrast enhanced FDG PET/CT, acquired in portal phase. Hence as discussed previously, results of our study suggest a relatively higher threshold values as a result of effect of IV contrast media on bone attenuation values.

 Comparison and analysis of mean VHU and FNHU values with respective DXA T-scores using Bland Altman plots**)** and Passing and Bablok regression analysis revealed a high degree of correlation between two modalities for detection of osteopenia and osteoporosis with a statistically insignificant bias of 0.24 and p value <0.0001.

 Our results also showed that younger women had higher bone attenuation and corresponding higher SUV_max_ values as compared to post-menopausal women. In older women bone marrow fat proportion increases, which is metabolically less active than hematopoietic marrow, and hence there is decrease in SUV_max_ in these women (39, 40). 

 Our study demonstrated a post chemo ± hormonal therapy mean decline of ~14% in vertebral and femoral neck mean SUV_max_ values in post chemo ± hormonal therapy patients, compared to reduction of 11.5% in reported in study by Alobthani (41). Fatty and fibrotic marrow changes with decreased bone osteoblastic cell activity is also thought to occur in post chemo ± hormonal therapy patients (42-44). Results from our study may provide some evidence that reduced bone marrow metabolism as assessed by FDG SUV_max_ values could possibly indirectly predict decreased bone mineral density. Here it is worth noting that in patients treated with chemotherapy and colony stimulating factor, initial observed increase in bone marrow SUV_max_ usually returns to pretreatment levels approximately 1 month after discontinuation of treatment (45). 

 However, SUV_max_ measurements should be interpreted with caution and in correlation with CECT attenuation values as they may be influenced by multiple confounding factors discussion of which is beyond the scope of this paper.

 Our study suggested a balanced threshold of L1-L4 mean attenuation ≤174.6 HU for osteopenia, and ≤117.2 HU for osteoporosis with sensitivity and specificity of 100% and 98.4%, whereas femoral neck mean attenuation ≤176.8 HU for osteopenia and ≤117.8 HU for osteoporosis with sensitivity and specificity of 100% and 98.4% respectively on contract enhanced PET/CT scans. Furthermore, threshold mean attenuation values of ≤125.9 HU with sensitivity and specificity of ~100% and 79% and ≤124.8 HU with sensitivity of 100% and specificity ~79.8% were suggested for increased L1-L4 vertebral and femoral neck fractures prevalence/ risk respectively. Relative risk and Odd’s ratio for post chemo ± hormonal therapy vertebral fractures were 1.8 and 1.87 whereas for femoral neck fractures respective values were at 2.3 and 2.42 with P values <.0.001.

 Since there can be a significant discrepancy between mean attenuation values of lumbar vertebrae and femoral necks with lumbar vertebrae being osteopenic while femora having normal attenuation or vice versa therefore measurement of both mean VHU and FNHU on portal phase of CECT component of routine oncologic FDG PET/CT supported by SUV_max_ values allow a simple, fast, convenient and inexpensive detection of patients with low bone mineral density prior to chemotherapy, and as after completion of therapy thus identifying patients with osteoporosis, at risk for developing osteoporosis or osteoporotic fractures. This could reduce need for DXA scans, reducing the costs and enable early and appropriate therapeutic interventions such as calcium and Vitamin D supplementation, bisphosphonates (46, 47) or more recently human monoclonal antibody RANKL inhibitor Denosumab (48), as required. Further, this semi-quantitative method requires negligible amount of training and time; could be applied prospectively or retrospectively with no additional cost; no additional patient time, no specialized equipment or radiation exposure (13). It also offers a significant advantage over methods, such as quantitative CT which is more labor intensive and requires an asynchronous calibration process (13). 

**Figure 15:**
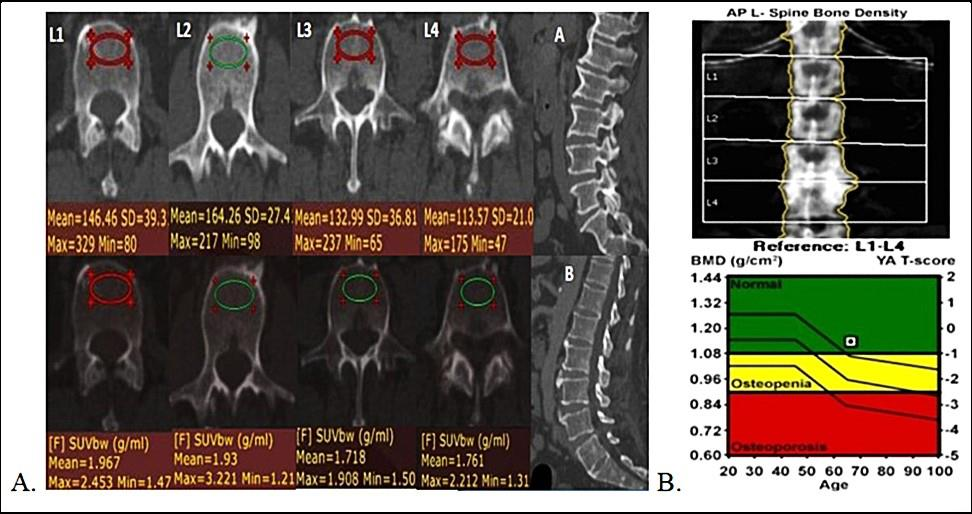
**A.** Initial staging axial PET/CT images in a 67 year old female with IDC breast carcinoma in top row show reduced VHU with degenerative osteophytes (L1-L4 mean HU of 139). Fused axial PET/CT images in bottom row show SUV_max_ values of L1-L4 vertebrae. However no fractures were seen. Sagittal CECT images (**A **and** B**) also show marked degenerative changes in the spine.** B. **DXA scan results of this patient show mean L1-L4 T- scores -0.4, above the cutoff limit of < -1.0 used to define osteopenia on DXA scans. This is a case of false negative DXA scan overestimating T - scores because of degenerative changes

**Figure 16:**
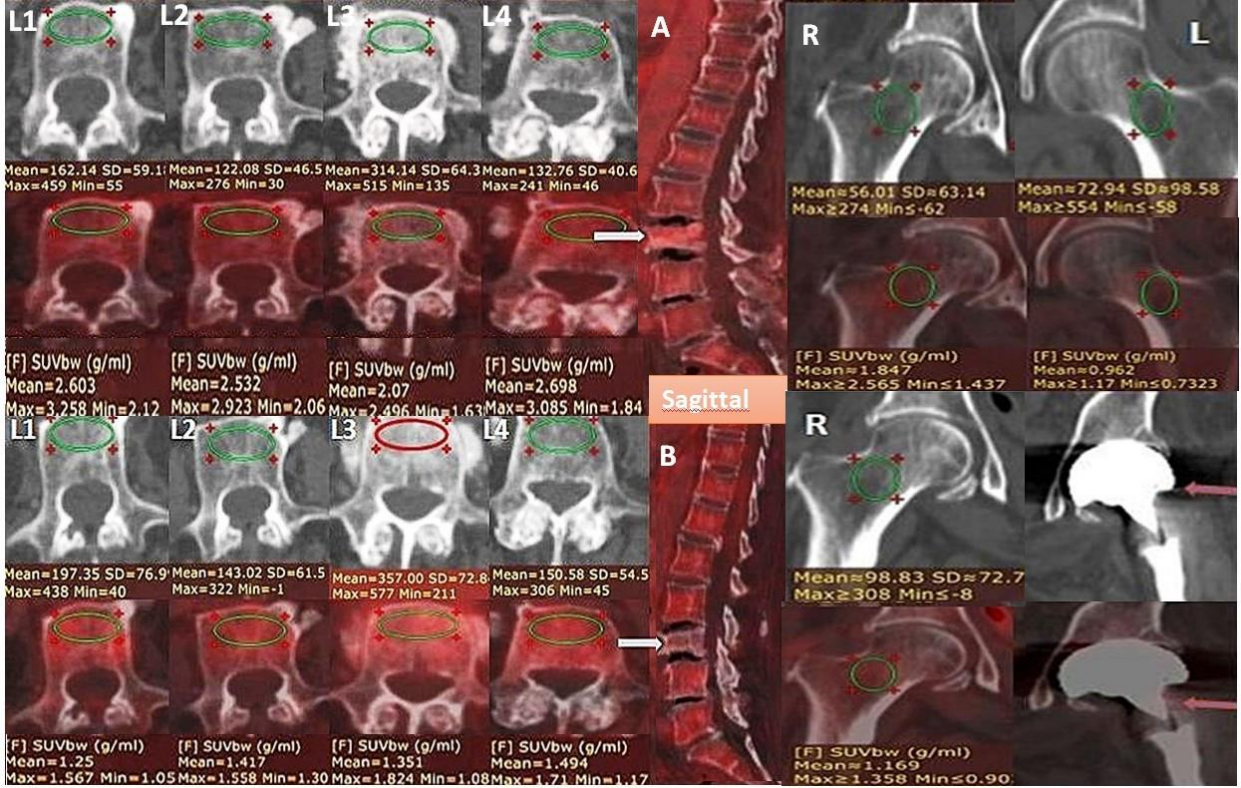
Initial staging (top rows) and post treatment axial PET/CT images (bottom rows) in a 66 year old female. Pretreatment CECT (top row) shows reduced VHU of L1, L2 and L4 with increase attenuation and sclerosis at L3. Sagittal images** (A **and** B)** shows compression/collapse of L3 vertebra (**white arrow**). (R and L in top rows) also show reduced bilateral FNHU. L1-L4 mean HU is 182.7 which is erronously high because of abnormal false high attenuation value caused by collapsed L3 vertebra, while mean L1-L4 SUV_max_ is 2.91; Mean FNHU and mean SUV are 64.5 HU and 1.86 respectively). Post treatment follow up PET/CT images (Bottom rows) 8 months later shows increase in attenuation of corresponding vertebrae with minimally reduced SUV_max_ values (L1-L4 mean HU ~ 212; and mean SUV_ma_x 1.66; an overall increase of ~11% in mean VHU and ~10% decrease in mean SUV_max_; whereas right femoral neck CT attenuation got increased by 75% and SUV_max_ declined by 47% respectively). In addition, on follow up scan, fracture of left femoral neck was also noted with left unipolar hemiarthroplasty. This indicates that conventional bisphophonates may not be very effective in preventing osteoporotic fractures and therefore newer drugs like RANKL inhibitor human monoclonal antibody Denosumab which has shown superior results in prevention of osteoporotic fractures may be preferred in severe osteoporosis. Comparative DXA scans were not available

**Figure 17:**
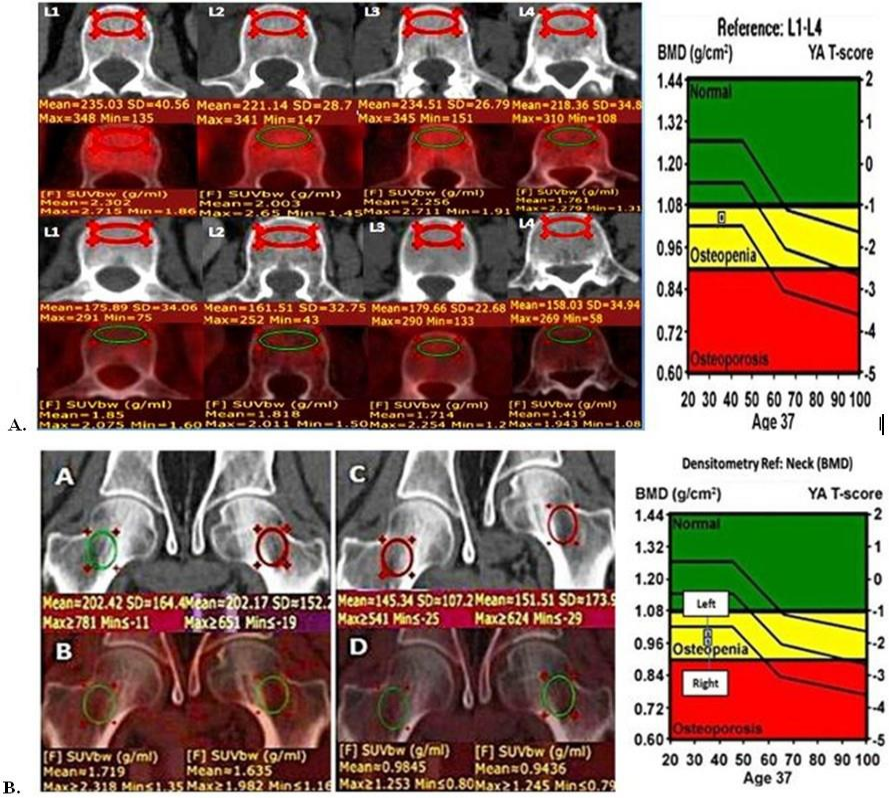
A. Initial staging (top rows) and post Chemo ± hormonal therapy (bottom rows) axial PET/CT images in a 37 year old female with IDC breast. Initial Staging PET/CT show normal L1 to L4 attenuation values (L1- L4 mean HU of 227) while post Chemo ± hormonal therapy scan revealed reduced lumbar vertebral attenuation (L1- L4 mean HU ~ 168 HU); an overall decline of 26%. Corresponding mean L1-L4 SUV_max_ values also demonstrated a decline from 2.58 to 2.04 ~ 20% decline. Post Chemo ± hormonal therapy DXA scan L1- L4 mean T- score was -1.3. B. Mean FNHU declined from 202.45 to 148.4 HU, a decline of ~ 54%, while mean L1-L4 SUV_max_ values also demonstrated a decline from 2.58 to 2.04 ~ 20% decline. Corresponding mean femoral neck T- score was -1.8

 Some examples of performance of pre and post chemo ± hormonal therapy FDG PET/CT scans compared to corresponding DXA data for evaluation of osteopenia, osteoporosis and detection of increased fracture risk are demonstrated and discussed in Figures 15-17. 

### Limitations of the studyIt was a retrospective study, performed in a single clinical institution with a relatively small study size. Since ROIs were placed manually, this non automated nature of attenuation and SUV measurement could make the measurements more variable. No separate subgrouping or classification of patient categories depending on type of chemotherapy combination or hormonal therapy administered was carried out. No particular assessment of dose or duration of steroids or duration of hormonal therapy was made. It is necessary to be cautious when interpreting these factors in this study. Finally DXA scans had not been performed in majority of pretreatment patients. Hence PET/CT attention values and DXA scan T- scores comparison was possible only in post chemo ± hormonal therapy treated patients.

## Conclusion

 Baseline and post chemo ± hormonal therapy follow up FDG PET/CT scans in breast carcinoma patients allow opportunistic screening and can identify a significant number of patients with osteopenia and patients at risk for osteoporosis and increased fracture risk with a high sensitivity and good specificity. They have potential to reduce need for DXA referrals, and also enable early initiation of prophylasix and therapy in such patients. Moreover they have statistically strong Spearman’s rank correlation coefficients with DXA scan T- scores. The study also demonstrated a significant decline in post chemo ± hormonal therapy vertebral and femoral neck mean SUV_max_ values, which may serve as surrogate/indirect marker for decreased BMD but should be interpreted with caution and in correlation in CT attenuation values as SUV_max_ is influenced by many confounding factors. Hence we conclude that use of FDG PET/CT for opportunistic screening for osteopenia and osteoporosis have potential to reduce the need for DXA referrals, also enable early initiation of prophylaxis and therapy to prevent fractures in such patients. Therefore treating oncologists should seek or demand this parameter from both pre and post treatment FDG/PET scans.

## List of abbreviations used

**BMD:** Bone Mineral Density

**NCCT:** Non Contrast enhanced Computed Tomography

**CECT:** Contrast Enhanced Computed Tomography

**HU:** Hounsfield Unit

**VHU:** Vertebral Hounsfield Unit

**FNHU:** Femoral Neck Hounsfield Unit

**PET:** Positron Emission Tomography

^18^F-FDG: ^18^Fluorine-Fluorodeoxyglucose

**SUV**_max_ **:** Maximum Standardized Uptake Value

**DXA:** Dual-energy X-ray Absorptiometry

**ROC:** Receiver Operating Characteristic

**AUC:** Area Under the Curve

**ROI:** Region Of Interest

**SD:** Standard Deviations

**C.I:** Confidence Interval
